# Health Benefits and Molecular Mechanisms of Urolithin A: From a Gut Microbiota‐Derived Metabolite to Translational Applications

**DOI:** 10.1002/fsn3.72167

**Published:** 2026-07-24

**Authors:** Zijiang Yang, Chenggen Guo, Ziao Deng, Xinxuan Xue

**Affiliations:** ^1^ School of Art and Physical Education Nanchang Jiaotong Institute Nanchang Jiangxi China; ^2^ School of Sports Training Wuhan Sports University Wuhan Hubei China; ^3^ Department of Linguistics Law & Business College of Hubei University of Economics Wuhan Hubei China

**Keywords:** aging‐related diseases, clinical translation, gut microbiota, mitophagy, Urolithin A

## Abstract

Urolithin A (UA) is an important bioactive metabolite generated by the gut microbiota from ellagic acid and ellagitannins. In recent years, it has attracted widespread attention because of its potential value in aging intervention and the prevention and treatment of multisystem diseases. This review systematically summarizes the molecular mechanisms of UA, its effects in disease intervention, and progress in human clinical studies. Existing studies have shown that, with mitochondrial quality control as the core, UA exerts multi‐target biological effects through the synergistic regulation of pathways related to inflammatory responses, oxidative stress, mitophagy, and programmed cell death. Findings from in vitro mechanistic studies and animal models suggest that UA may influence pathological processes relevant to degenerative musculoskeletal diseases, cardiovascular diseases, neurological disorders, and cancers, whereas current human clinical evidence remains preliminary and is mainly concentrated in muscle function, exercise‐related outcomes, selected metabolic biomarkers, and short‐ to medium‐term safety and tolerability. In addition, this review discusses the bioavailability of UA and its relationship with individual gut microbiota metabolic phenotypes. Future research should focus on high‐quality, long‐term randomized controlled trials with extended follow‐up to promote the application of UA in precision nutrition and its clinical translation.

## Graphical abstract

Urolithin A (UA) is a gut microbiota‐derived metabolite produced from dietary ellagitannins and ellagic acid, with endogenous formation varying among urolithin metabotypes (UM‐A, UM‐B, and UM‐0). UA regulates an interconnected network centered on mitophagy and mitochondrial quality control, antioxidant defense, inflammatory signaling, autophagy, and cell survival. Preclinical evidence suggests potential protective effects in degenerative musculoskeletal, cardiovascular, neurological, and cancer‐related conditions, while emerging human studies support possible roles in skeletal muscle function, exercise adaptation, and recovery. Direct oral supplementation may reduce variability associated with microbiota‐dependent UA production; however, larger, long‐term clinical trials are required to establish efficacy, optimal dosing, responder profiles, and long‐term safety.

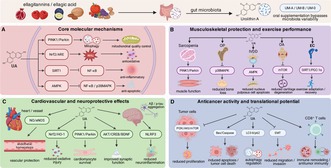

## 1 Introduction

Urolithin A (UA) is a metabolite produced in the colon by the gut microbiota through the conversion of the natural polyphenols ellagitannins and ellagic acid. These polyphenols are widely found in foods such as pomegranates, strawberries, raspberries, and walnuts (Ribeiro et al. 2025). UA was first identified in rats by Doyle in 1980 (D'Amico et al. 2021). The chemical structure of UA is characterized by an α‐benzocoumarin skeleton. Its molecular formula is C13H8O4, and its molecular weight is 228.20 g/mol. Its core structure is a tricyclic fused aromatic compound containing a lactone ring, which confers high stability and forms the basis of its biological activity (Hasheminezhad et al. 2022).

UA has been widely studied because of its multiple biological effects. It possesses anti‐inflammatory, antioxidant, and antiapoptotic properties, and has shown particular potential in promoting mitophagy, maintaining mitochondrial function, and preserving cellular metabolic homeostasis (Borsky et al. 2025). These multi‐target regulatory mechanisms suggest that UA provides a biological rationale for further investigation in degenerative skeletal muscle diseases, cardiovascular diseases, neurological disorders, and malignant tumors. However, the in vivo production of UA is highly dependent on the conversion capacity of an individual's gut microbiota, and individuals can be classified into different metabotypes, such as UM‐A, UM‐B, and UM‐0 (Pidgeon et al. 2025). In addition, once UA enters the bloodstream, it mainly exists as phase II conjugates, while free UA remains at low levels. Together, these factors increase inter‐individual variability in response and the uncertainty of extrapolating findings to clinical settings (Zhang, Cui, et al. 2023).

Against this background, this review focuses on the molecular mechanisms of action of UA and its potential health benefits, summarizing its specific targets involved in the regulation of inflammatory signaling pathways, the alleviation of oxidative stress, the maintenance of mitochondrial quality control, and the inhibition of programmed cell death. Meanwhile, this review also systematically summarizes the latest research advances on UA in degenerative skeletal muscle diseases, cardiovascular diseases, neurological disorders, and cancer, and further discusses research trends regarding its use as a sports nutrition supplement. Finally, this paper reviews key issues such as the bioavailability, clinical evidence in humans, and safety of UA, with the aim of informing future scientific research and drug development involving this natural metabolite.

## 2 Molecular Mechanisms of UA

**FIGURE 1:**
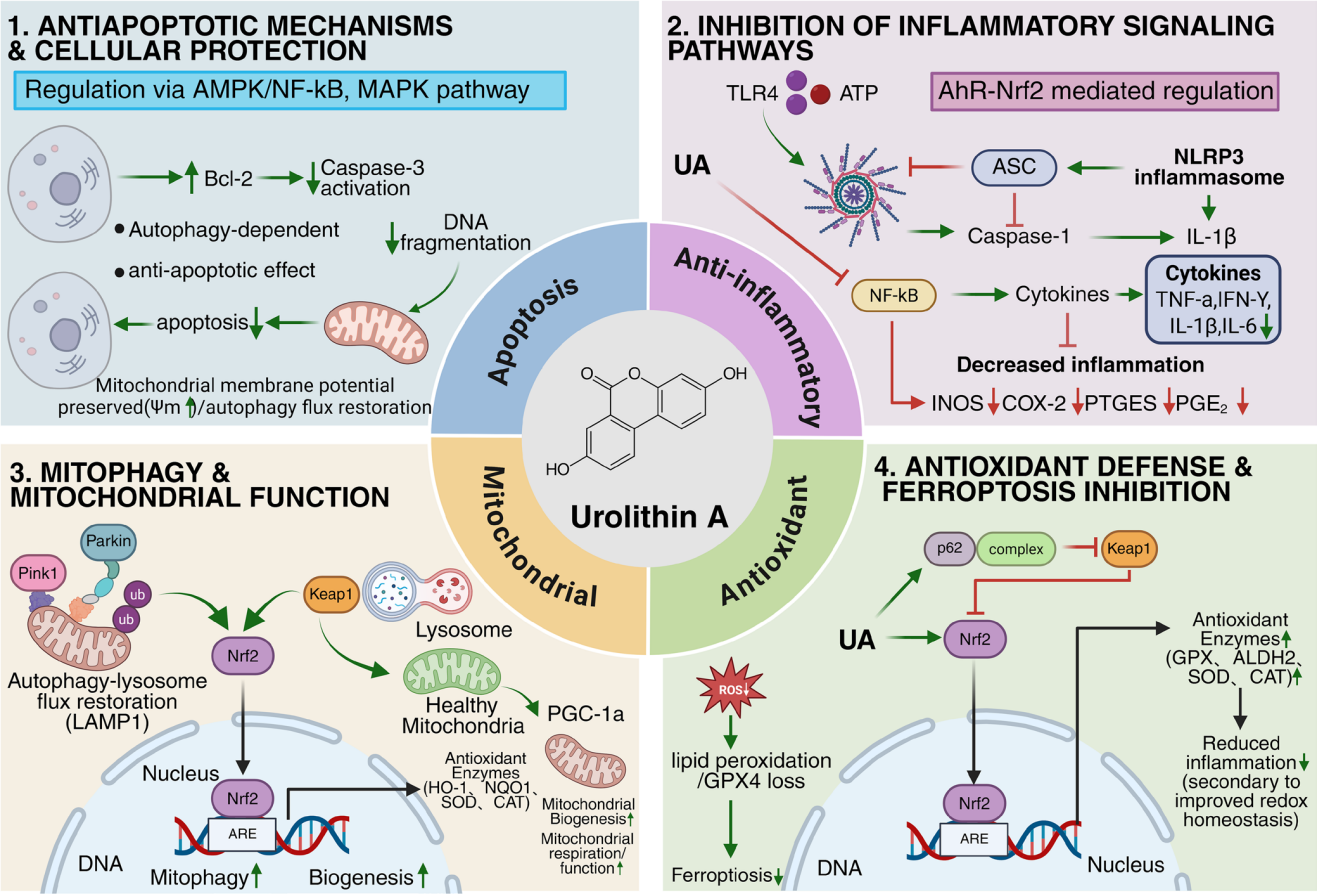
Molecular mechanisms of UA. This figure summarizes the major molecular mechanisms through which UA may exert cytoprotective effects across different disease models. These mechanisms are organized into four functional categories: Antiapoptotic mechanisms and cellular protection, inhibition of inflammatory signaling pathways, mitophagy and mitochondrial function, and antioxidant defense and ferroptosis inhibition. UA may reduce apoptosis by regulating AMPK/NF‐κB and MAPK‐related pathways, increasing Bcl‐2 expression, decreasing caspase‐3 activation and DNA fragmentation, preserving mitochondrial membrane potential, and restoring autophagy flux. UA may also attenuate inflammatory responses through modulation of TLR4/NF‐κB, AhR‐Nrf2, and NLRP3 inflammasome‐related signaling, leading to reduced cytokine production and decreased expression of inflammatory mediators such as iNOS, COX‐2, PTGES, and PGE2. In addition, UA may enhance PINK1/Parkin‐dependent mitophagy, autophagy–lysosome flux, Nrf2 nuclear translocation, mitochondrial biogenesis, and mitochondrial respiratory function. UA‐related activation of the p62‐Keap1‐Nrf2/ARE axis may further increase antioxidant enzyme expression, reduce ROS accumulation and lipid peroxidation, and inhibit ferroptosis.

Current evidence suggests that UA exerts cytoprotective effects across various disease conditions by regulating key processes such as inflammatory responses, oxidative stress, mitochondrial homeostasis, and apoptosis (Sun et al. 2026; Yuan et al. 2026; L. Zhang et al. 2026). Its molecular mechanisms mainly involve multiple signaling pathways, including NF‐κB, Nrf2/ARE, PINK1/Parkin, and AMPK, and are characterized by multi‐level, networked regulation (Figure 1). Because much of the mechanistic evidence is derived from cellular and animal models, these pathways should be interpreted as putative and context‐dependent mechanisms rather than established therapeutic mechanisms in humans.

### 2.1 Regulation of Inflammatory Pathways

UA suppresses inflammatory responses through multiple signaling pathways, and its anti‐inflammatory potential was first identified in a dextran sulfate sodium (DSS)‐induced rat model of colitis. Studies have shown that UA effectively reduces the levels of inflammatory markers, including iNOS, COX‐2, PTGES, and PGE_2_, while also exerting beneficial effects on the gut microbiota (Larrosa et al. 2010). Subsequent studies further indicated that UA inhibits the production of inflammatory cytokines, such as TNF‐α, IFN‐γ, IL‐1β, and IL‐6, through activation of the AhR–Nrf2 pathway, suggesting its potential in the treatment of inflammatory diseases such as inflammatory bowel disease (IBD) (R. Singh et al. 2019). Notably, in a severe acute pancreatitis (SAP) rat model, UA improved both local pancreatic and systemic inflammatory conditions by regulating endoplasmic reticulum–mitochondrial calcium channels and inhibiting necroptosis‐related proteins, thereby exerting anti‐inflammatory effects (Kang et al. 2024). In addition, UA suppresses NF‐κB pathway activation through multiple mechanisms, including SIRT1 activation and inhibition of the phosphorylation of p65 and IκB proteins (Abdelazeem et al. 2021; N. Ghosh et al. 2020; X. He et al. 2024).

These findings indicate that the anti‐inflammatory activity of UA is not limited to reducing cytokine release, but involves regulation of several molecular events upstream and downstream of inflammatory signaling. In particular, suppression of NF‐κB activation, reduced phosphorylation of p65 and IκB, modulation of AhR–Nrf2 signaling, and regulation of endoplasmic reticulum–mitochondrial calcium channels suggest that UA may attenuate inflammation by acting on interconnected stress‐response and cytokine‐regulatory nodes.

### 2.2 Antioxidant Properties

At appropriate doses, UA plays an important role in alleviating oxidative damage. In a mouse model of renal ischemia–reperfusion‐induced acute kidney injury (AKI), UA activated the p62–Keap1/Nrf2 pathway, enhanced the expression of antioxidant enzymes such as superoxide dismutase and catalase, and reduced ROS levels (Y. Zhang et al. 2022). Similar effects were also observed in a mouse model of acetaminophen‐induced acute liver injury, in which UA alleviated glutathione depletion and lipid peroxidation through the Nrf2/ARE pathway, with efficacy superior to that of the commonly used clinical drug N‐acetylcysteine (Gao et al. 2022). In a rotenone‐induced rat model of Parkinson's disease (PD), UA reduced ROS and their harmful products, including MDA and 4‐HNE, increased the activities of ALDH2 and GPx, and protected neurons against oxidative damage by inhibiting α‐synuclein aggregation (Kujawska et al. 2019). In addition, UA exerted antioxidant effects in a lipopolysaccharide (LPS)‐induced mouse model of acute lung injury by activating the Keap1–Nrf2/HO‐1 pathway, thereby suppressing ferroptosis‐associated lipid peroxidation and GPX4 loss.

The available evidence supports Nrf2‐centered redox regulation as a major molecular basis for the antioxidant effects of UA. By activating p62–Keap1/Nrf2, Nrf2/ARE, and Keap1–Nrf2/HO‐1 signaling, UA appears to enhance endogenous antioxidant defenses, preserve glutathione‐related redox balance, and reduce ROS accumulation, lipid peroxidation, and ferroptosis‐associated oxidative injury across different experimental models.

### 2.3 Mitochondrial Function

Mitochondria are dynamic organelles that are essential for maintaining cellular homeostasis and determining cell survival or death (Zhang, Gao, Zhang, et al. 2025). By metabolizing glucose, fatty acids, and amino acids through oxidative pathways, mitochondria generate ATP to support normal cellular activities. Beyond energy production, they are also involved in the regulation of apoptosis, calcium homeostasis, immune signaling, and cellular redox status (Monzel et al. 2023). Evidence from cellular, nematode, murine, and human studies indicates that one of the most consistent effects of UA is the improvement of mitochondrial health through mitophagy (Faitg et al. 2024). In *Caenorhabditis elegans* , UA initiates PINK1/Parkin‐mediated mitophagy by reducing mitochondrial membrane potential, thereby promoting the clearance of damaged mitochondria and inducing mitochondrial biogenesis. These effects help maintain and enhance mitochondrial respiration, improve skeletal muscle function, and delay age‐related mitochondrial decline (Ryu et al. 2016). In a transgenic tau‐expressing *C. elegans* model, UA reduced tau hyperphosphorylation through the mitophagy pathway, thereby improving synaptic function and cognitive performance (Fang et al. 2019). In a Duchenne muscular dystrophy (DMD) mouse model, UA increased the expression of mitophagy‐related markers such as LC3 and p62, enhanced autophagic flux, and accelerated the clearance of damaged mitochondria (Luan et al. 2021). Moreover, in an amyotrophic lateral sclerosis (ALS) mouse model, UA increased the expression of PINK1, Parkin, and LAMP1, and restored the function of the autophagy–lysosome pathway (Zhang, Gao, Yang, et al. 2025). Clinical studies have shown that supplementation with UA reduces plasma acylcarnitine levels in older adults, suggesting an improvement in mitochondrial fatty acid oxidation and energy metabolism efficiency (Liu, D'Amico, et al. 2022). Notably, a recent clinical study in elite athletes showed that UA enhanced Parkin phosphorylation, thereby promoting the ubiquitination of mitochondrial outer membrane proteins, autophagosome formation, and subsequent lysosomal degradation. In addition, proteomic and gene set enrichment analysis (GSEA) results indicated that UA supplementation significantly enriched proteins associated with mitochondrial protein complexes, organelles, and mitochondrial ribosomes, further supporting the activation of mitochondrial biogenesis (Whitfield et al. 2025).

Compared with other mechanisms, mitochondrial quality control appears to be a particularly consistent molecular function of UA. Evidence from cellular, nematode, murine, and human studies suggests that UA promotes the removal of damaged mitochondria through PINK1/Parkin‐mediated mitophagy and restoration of autophagy–lysosome activity, while also supporting mitochondrial biogenesis, fatty acid oxidation, and mitochondrial protein complex enrichment. These findings place mitophagy and mitochondrial homeostasis at the center of UA‐related molecular regulation.

### 2.4 Antiapoptotic Effects

Apoptosis, also known as programmed cell death, is a fundamental defense mechanism by which the body eliminates infected or damaged cells (Zhan et al. 2024). Existing studies have shown that UA exerts antiapoptotic effects in multiple disease models. In a cisplatin‐induced mouse model of nephrotoxicity, UA reduced caspase‐3 activity and DNA fragmentation, thereby alleviating renal cell apoptosis (Jing et al. 2019). In a mouse model of cerebral ischemic injury, UA exerted neuroprotective effects by upregulating Bcl‐2 expression and modulating the AMPK/NF‐κB and MAPK signaling pathways, thereby inhibiting neuronal apoptosis (Lin, Ye, et al. 2020). Similarly, in an amyloid precursor protein/presenilin 1 (APP/PS1) mouse model, UA also showed antiapoptotic activity. It alleviated neuronal injury and reduced apoptosis by promoting hippocampal neurogenesis, activating the AMPK pathway, and inhibiting the NF‐κB and p38MAPK inflammatory signaling pathways (Gong et al. 2019). In addition, in a type 2 diabetes model consisting of high‐fat diet/streptozotocin (HFD/STZ)‐induced mice and MIN6 pancreatic β‐cells, UA attenuated glucolipotoxicity‐induced β‐cell apoptosis by inhibiting caspase activation, preserving mitochondrial membrane potential, restoring autophagic flux, and exerting autophagy‐dependent antiapoptotic effects (Y. Zhang et al. 2021).

The antiapoptotic effects of UA seem to be closely connected with its ability to preserve mitochondrial function and regulate stress‐related survival pathways. Across different injury models, UA reduced caspase‐3 activity and DNA fragmentation, increased Bcl‐2 expression, maintained mitochondrial membrane potential, and restored autophagic flux, while AMPK, NF‐κB, MAPK, and p38 MAPK signaling may link mitochondrial stress, inflammation, and apoptosis‐related responses.

Taken together, these mechanistic studies suggest that UA does not act through isolated signaling pathways, but may regulate an interconnected molecular network centered on mitochondrial quality control, redox balance, inflammatory signaling, and cell survival. Among these mechanisms, mitochondrial quality control may represent a central functional node, because improved mitophagy and mitochondrial homeostasis can reduce ROS accumulation, limit NF‐κB‐mediated inflammatory activation, preserve cellular energy metabolism, and attenuate mitochondria‐dependent apoptosis. Conversely, persistent inflammation and oxidative injury may further impair mitochondrial function, creating a pathological feedback loop that UA may partly interrupt. Therefore, the putative molecular targets of UA should be interpreted as a context‐dependent regulatory network involving PINK1/Parkin, AMPK/SIRT1/PGC‐1α, p62–Keap1/Nrf2, NF‐κB, MAPK, PI3K/AKT/mTOR, and apoptosis‐related mediators such as Bcl‐2 family proteins and caspases. Nevertheless, the relative contribution of these pathways is likely to vary across disease models, cell types, doses, and intervention durations. Further studies are needed to distinguish direct molecular targets from downstream adaptive responses and to determine which mechanisms are most relevant under clinically achievable exposure conditions.

## 3 Health Benefits of UA

### 3.1 Degenerative Musculoskeletal Diseases

**FIGURE 2:**
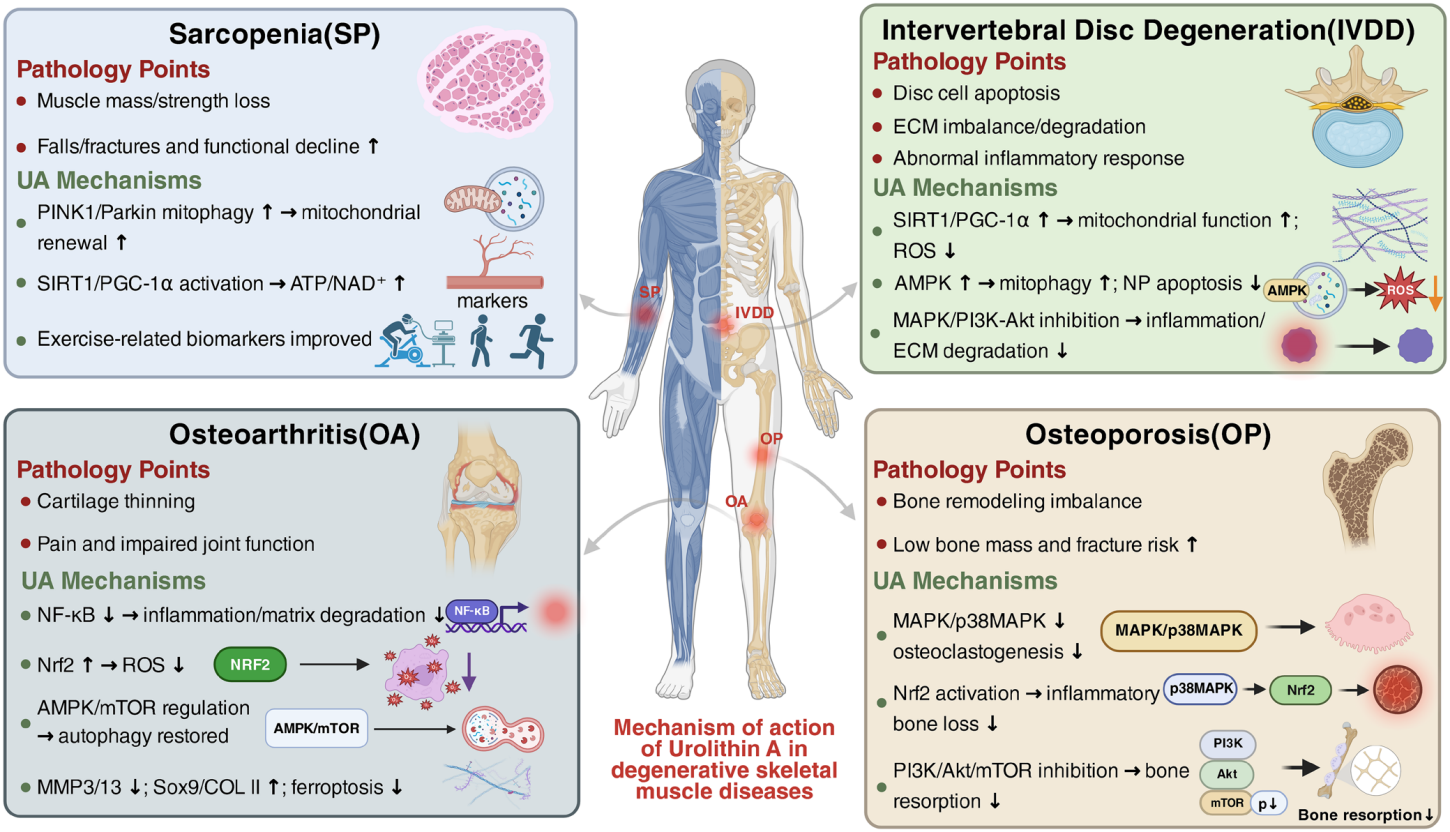
Potential protective mechanisms of UA in degenerative musculoskeletal diseases. This figure summarizes the major pathological features and proposed protective mechanisms of UA in four representative degenerative musculoskeletal diseases, including sarcopenia (SP), intervertebral disc degeneration (IVDD), osteoarthritis (OA), and osteoporosis (OP). The central schematic highlights the representative anatomical sites involved in each condition. In sarcopenia, UA may enhance PINK1/Parkin‐dependent mitophagy and SIRT1/PGC‐1α signaling, thereby promoting mitochondrial renewal, improving ATP/NAD+ metabolism, and contributing to improved exercise‐related biomarkers. In IVDD, UA may activate SIRT1/PGC‐1α‐ and AMPK‐related pathways to improve mitochondrial function, promote mitophagy, reduce ROS accumulation, suppress nucleus pulposus cell apoptosis, and attenuate inflammation and extracellular matrix (ECM) degradation through inhibition of MAPK and PI3K/Akt signaling. In OA, UA may suppress NF‐κB‐mediated inflammation and matrix degradation, activate Nrf2‐related antioxidant responses to reduce ROS, regulate AMPK/mTOR‐associated autophagy, decrease MMP3/MMP13 expression, increase Sox9 and collagen II expression, and inhibit ferroptosis. In OP, UA may suppress MAPK/p38MAPK signaling and osteoclastogenesis, activate Nrf2‐related protective responses to reduce inflammatory bone loss, and inhibit PI3K/Akt/mTOR signaling to attenuate bone resorption.

Degenerative musculoskeletal diseases are an important cause of disability worldwide, mainly including osteoarthritis (OA), osteoporosis (OP), intervertebral disc degeneration (IVDD), and sarcopenia (SP) (Mao et al. 2025). Existing studies suggest that UA may influence the progression of these diseases through mechanisms related to inflammation, oxidative stress, autophagy, and mitochondrial quality control (Figure 2).

#### 3.1.1 Osteoarthritis (OA)

Osteoarthritis (OA) is a joint disease characterized by progressive cartilage damage and is associated with pain, functional impairment, and reduced quality of life (N. Fuggle et al. 2025). In chondrocyte models, UA alleviated cartilage damage induced by excessive mechanical loading by inhibiting chondrocyte senescence, restoring autophagy and mitochondrial function, upregulating NRF2 to enhance antioxidant defense, and reducing ROS and inflammatory cytokine levels, thereby suggesting that UA‐related pathways may represent experimental targets for further OA research (Y. He et al. 2021). For example, in a mouse OA model, UA significantly improved articular cartilage damage and delayed OA progression by inhibiting the NF‐κB pathway, reducing inflammatory responses, decreasing MMP‐mediated cartilage matrix degradation, increasing GSH and GPX4 to suppress ferroptosis, and activating AMPK while inhibiting mTOR to restore autophagy and energy homeostasis (Wang, Xu, et al. 2025). In a rat articular chondrocyte model, UA also ameliorated OA‐related cartilage damage by inhibiting IL‐1β‐mediated inflammatory responses (iNOS, COX2, NO, and PGE2), suppressing matrix degradation (MMP3 and MMP13), restoring cartilage synthetic capacity (Sox‐9 and Collagen II), and blocking the MAPK and NF‐κB signaling pathways (Ding et al. 2020). Collectively, these findings suggest that UA may protect cartilage through a coordinated mechanism involving inflammation suppression, oxidative stress reduction, ferroptosis inhibition, and restoration of autophagy, rather than through a single isolated pathway.

#### 3.1.2 Osteoporosis (OP)

Osteoporosis (OP) is a skeletal disorder caused by disrupted bone remodeling and is characterized by decreased bone mass and deterioration of bone microarchitecture, ultimately leading to a substantially elevated risk of fracture (Fuggle et al. 2024). UA mainly attenuates OP progression by inhibiting osteoclast activity and differentiation. Specifically, UA inhibited RANKL‐induced osteoclastogenesis by suppressing the MAPK cascade. At the same time, UA enhanced the autophagic capacity of bone marrow‐derived precursor cells (BMMs), maintained cellular homeostasis, and exerted a protective regulatory effect on bone remodeling (Tao et al. 2022). In a lipopolysaccharide (LPS)‐induced mouse model, UA alleviated RANKL‐induced osteoclastogenesis by inhibiting the p38MAPK pathway and inducing Nrf2 nuclear translocation, suggesting its potential to mitigate inflammation‐induced bone loss and bone resorption (Wei et al. 2022). Similar anti‐inflammatory properties were also confirmed in OVX mice. UA attenuated NF‐κB–dependent activation of the NOD‐like receptor signaling pathway, thereby reducing the secretion of IL‐1β and IL‐18 in the cytoplasm and decreasing the expression of pyroptosis‐related markers (NLRP3, GSDMD, and caspase‐1) (Tao et al. 2021). In addition, UA markedly inhibited the phosphorylation of PI3K, AKT, and mTOR, a signaling axis that acts as a key downstream regulator of bone resorption in the RANKL–TRAF6 pathway (Zhou et al. 2024).

#### 3.1.3 Intervertebral Disc Degeneration (IVDD)

Intervertebral disc degeneration (IVDD) is a major chronic disorder in spinal surgery and plays an important role in the pathogenesis of various degenerative spinal diseases (Q. Xia et al. 2024). Its main features include increased apoptosis of disc cells, an imbalance between extracellular matrix (ECM) anabolism and catabolism, and abnormal inflammatory responses (Lin et al. 2023). Current studies generally suggest that ECM degradation and loss of nucleus pulposus cells are important factors that accelerate IVDD progression (Vergroesen et al. 2015). UA exerts anti‐degenerative effects on intervertebral discs by activating the SIRT1/PGC‐1α signaling pathway, thereby improving mitochondrial function, reducing oxidative stress, and delaying nucleus pulposus cell senescence and ECM degradation (Shi et al. 2021). In H_2_O_2_‐induced rat nucleus pulposus cells, UA reduced TNF‐α‐induced inflammatory responses and ECM degradation by inhibiting the activation of the MAPK and PI3K/Akt signaling pathways (H. Liu et al. 2018). UA upregulated AMPK and activated mitophagy, thereby counteracting oxidative stress‐induced apoptosis in nucleus pulposus cells (Lin, Zhuge, et al. 2020). Although these findings identify several potential targets of UA in IVDD, the interactions among these pathways and their relative importance remain unclear.

#### 3.1.4 Sarcopenia (SP)

Sarcopenia (SP) is a progressive skeletal muscle condition marked by a decline in muscle strength and muscle mass, which in turn leads to falls, fractures, loss of independent living, and increased hospitalization rates. It represents an important global health burden and mainly affects older adults (Beaudart et al. 2025). In *C. elegans* and mouse models, UA activated PINK1/Parkin‐dependent mitophagy, promoted mitochondrial renewal and oxidative phosphorylation, and thereby improved muscle cell metabolism and energy supply (Ryu et al. 2016). In wild‐type rodents and Duchenne muscular dystrophy (mdx) mouse models, UA significantly upregulated the expression of mitophagy‐related markers in skeletal muscle tissue, such as Parkin, ubiquitination, and phosphorylated ubiquitin (Luan et al. 2021). In addition, after 16 weeks of oral administration in middle‐aged mice, UA promoted the expression of angiogenesis‐related markers in skeletal muscle, activated the SIRT1–PGC‐1α pathway, and increased ATP and NAD^+^ levels (N. Ghosh et al. 2020). Nanotherapy aims to deliver drug molecules to diseased sites more efficiently. A recent study showed that the nanoformulation MACL@UA significantly increased quadriceps muscle mass and angiogenesis in a rat model of systemic sarcopenia, which was attributed to the regulation of macrophage–muscle satellite cell metabolic interactions and mitochondrial homeostasis by UA (Zhang, Zhang, Zhu, et al. 2025). In terms of clinical research, randomized controlled trials have evaluated the potential of UA to support muscle function in middle‐aged and older adults. A randomized controlled trial in middle‐aged and older adults with relatively high BMI found that UA significantly increased peak oxygen consumption and six‐minute walk distance, while reducing C‐reactive protein and acylcarnitine levels, suggesting that UA may counteract muscle atrophy by alleviating chronic inflammation and improving mitochondrial efficiency (Singh et al. 2022b). Another clinical study also found that, after 4 months of UA supplementation, ceramide (C16 and C18) levels were markedly decreased in adults aged 65–90 years (Liu, D'Amico, et al. 2022). Interestingly, neither of these studies included exercise intervention, suggesting that UA may improve muscle function independently at the molecular and metabolic levels. Compared with other musculoskeletal conditions, the evidence for UA in sarcopenia is more translationally advanced, as preclinical findings centered on mitophagy and mitochondrial metabolism are supported by clinical improvements in muscle‐related functional and metabolic outcomes.

Taken together, the available evidence suggests that mitochondrial quality control may represent the most consistent mechanistic theme across OA, OP, IVDD, and sarcopenia. Although inflammation, oxidative stress, and autophagy are involved in disease‐specific contexts, improvements in mitochondrial function and cellular energy homeostasis appear to be recurrent findings across different musculoskeletal tissues. Notably, among the conditions discussed, sarcopenia currently has the strongest translational support because beneficial effects have been observed not only in preclinical models but also in randomized clinical trials. Future studies should determine whether the mechanistic benefits observed in cartilage, bone, and intervertebral disc tissues can be translated into clinically meaningful outcomes.

### 3.2 Cardiovascular Diseases (CVDs)

**FIGURE 3:**
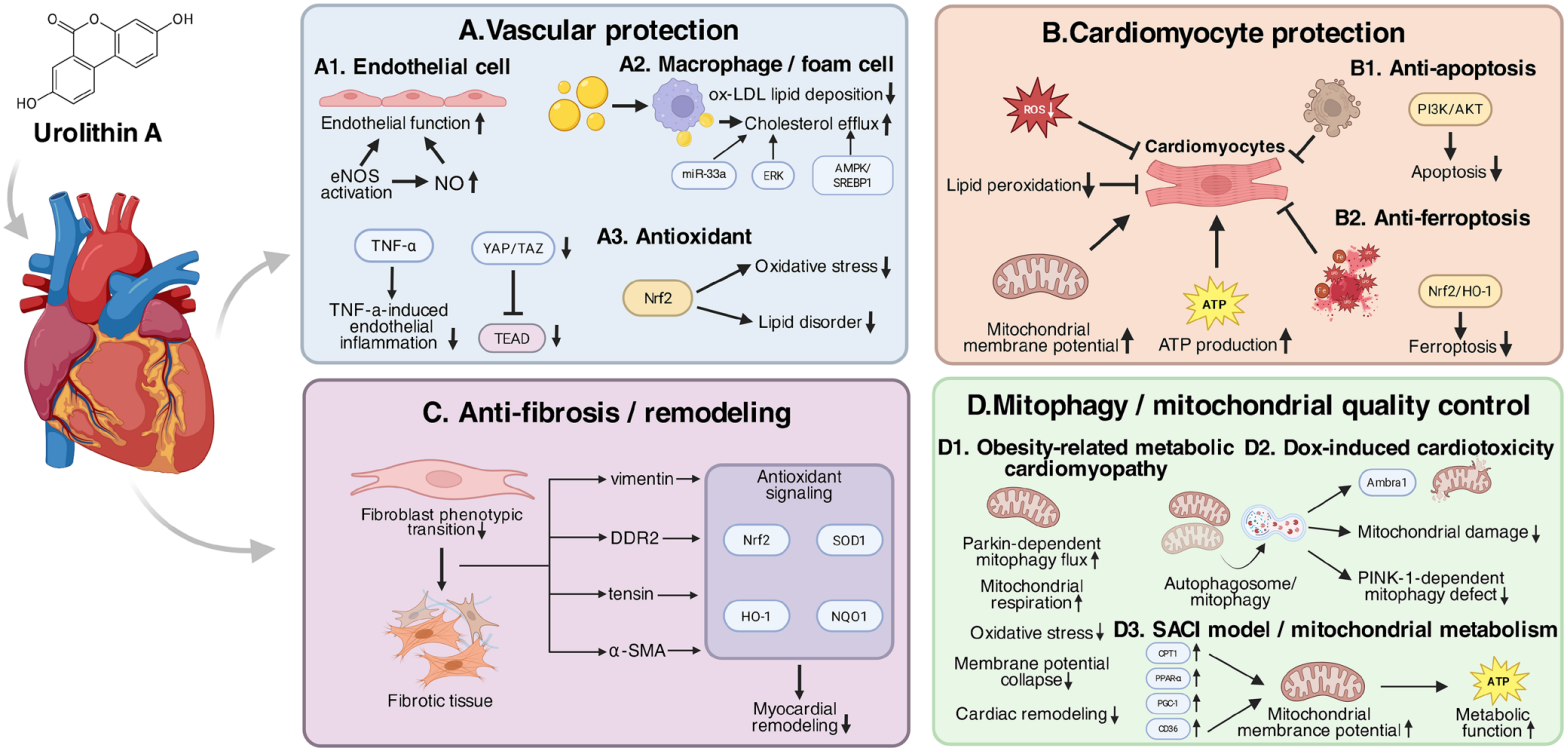
Potential cardiovascular protective mechanisms of UA. This figure summarizes the putative cardiovascular protective effects of UA across four major functional modules. (A) vascular protection: UA may improve vascular homeostasis by promoting endothelial function through eNOS/NO signaling (A1), regulating macrophage/foam cell lipid handling and cholesterol efflux through pathways involving miR‐33a, ERK, and AMPK/SREBP1 (A2), and reducing oxidative stress and lipid disorder through Nrf2‐related antioxidant responses (A3). (B) cardiomyocyte protection: UA may protect cardiomyocytes by attenuating ROS accumulation, lipid peroxidation, and mitochondrial dysfunction, while also suppressing apoptosis through PI3K/AKT signaling (B1) and ferroptosis through Nrf2/HO‐1 signaling (B2). (C) antifibrosis and cardiac remodeling: UA may reduce fibroblast phenotypic transition, fibrotic tissue formation, and myocardial remodeling through antioxidant and fibrosis‐related mediators, including Nrf2, SOD1, HO‐1, NQO1, DDR2, vimentin, tensin, and α‐SMA. (D) mitophagy and mitochondrial quality control: UA may improve mitochondrial quality control in different cardiac injury contexts, including obesity‐related metabolic cardiomyopathy (D1), doxorubicin‐induced cardiotoxicity (D2), and SAP‐associated acute cardiac injury‐related mitochondrial metabolism (D3).

Cardiovascular diseases (CVDs) remain a major global health burden because of their high incidence and mortality rates (Afshin et al. 2019). In this section, the available evidence is organized around vascular dysfunction, myocardial injury, cardiac fibrosis, and mitochondrial quality control in experimental cardiovascular models (Figure 3).

In terms of improving vascular function, UA primarily exerts its effects by inhibiting atherosclerosis and enhancing endothelial function. Basic research showed that UA pretreatment alleviated TNF‐α‐induced endothelial inflammation by promoting NO production, reducing YAP/TAZ protein expression, and decreasing TEAD transcriptional activity (M. Y. Xu et al. 2024). Furthermore, UA and its derivative metabolites significantly promoted the activation of eNOS and the release of NO in human aortic endothelial cells, suggesting that circulating urolithin metabolites may directly maintain endothelial homeostasis (Spigoni et al. 2016). In macrophages, UA inhibited ox‐LDL‐induced lipid deposition and promoted cholesterol efflux; its action is closely associated with the regulation of miR‐33a and the ERK/AMPK/SREBP1 signaling axis, thereby exerting a role in reshaping lipid metabolism in foam cells (Han et al. 2020). In a rat atherosclerosis model induced by high cholesterol and excessive vitamin D3, UA reduced local oxidative stress and lipid dysregulation through activation of Nrf2‐related antioxidant pathways (Cui et al. 2018). Collectively, these findings suggest that UA may support vascular homeostasis through coordinated regulation of endothelial NO signaling, inflammatory transcriptional activity, macrophage lipid handling, and antioxidant defense.

Regarding the improvement of myocardial function, UA primarily exerts its protective effects by maintaining mitochondrial functional homeostasis and inhibiting various forms of programmed cell death. In models of ischemia–reperfusion and hypoxia‐reoxygenation injury, UA reduced ROS production and lipid peroxidation, restored mitochondrial membrane potential and ATP production, and alleviated cardiomyocyte apoptosis. These effects were associated with PI3K/Akt signaling and the endogenous antioxidant system (Su et al. 2024; Tang et al. 2017). UA pretreatment also attenuated ferroptosis through the Nrf2‐HO‐1 pathway (Su et al. 2024). Similar intervention effects were also demonstrated in a TGF‐β1‐induced cardiac fibroblast model, where UA inhibited cardiac fibroblast‐to‐myofibroblast transition (CMT), downregulated fibrosis markers such as vimentin, DDR2, tensin, and α‐SMA, and restored the Nrf2/SOD1/HO‐1/NQO1 signaling network, thereby alleviating cardiac fibrosis (Chen et al. 2022).

UA has also been evaluated in models of metabolic cardiomyopathy, Dox‐induced cardiotoxicity, and SAP‐associated acute cardiac injury (SACI), in which mitochondrial quality control emerged as a recurring target. In an obesity‐associated metabolic cardiomyopathy model, UA improved cardiac diastolic function and reduced cardiac remodeling by restoring Parkin‐dependent mitochondrial autophagy flux, enhancing mitochondrial respiratory capacity, and alleviating oxidative stress and membrane potential collapse (J. R. Huang et al. 2023). Furthermore, UA alleviated Dox‐induced mitochondrial damage and PINK1‐dependent mitochondrial autophagy defects via the Ambra1 pathway, suggesting that UA intervention may effectively reverse Dox‐induced cardiotoxicity (Wang, Ma, et al. 2025). In the SACI mouse model, UA upregulated the expression of CPT1, PPAR‐α, PGC‐1α, and CD36, increased mitochondrial membrane potential and ATP production, and restored mitochondrial metabolic functions (Yang et al. 2023). Interestingly, the aforementioned studies also found that etomoxir (ETO) significantly suppressed CPT1 expression in SACI, while UA appeared unable to mitigate this effect, suggesting that UA's beneficial effects on myocardial metabolism may be highly dependent on CPT1‐mediated fatty acid oxidation flux. These observations indicate that UA‐related cardioprotection may depend not only on general mitochondrial protection but also on the preservation of specific metabolic fluxes, particularly fatty acid oxidation in metabolically stressed myocardium.

Human evidence regarding the cardiovascular effects of UA remains limited. In a recent clinical trial involving older adults, oral UA was well tolerated and reduced plasma ceramide levels, including ceramide species incorporated into cardiovascular risk scores (S. Liu et al. 2025). Whether these biomarker changes translate into a reduced risk of cardiovascular disease, however, remains to be established. Overall, preclinical studies suggest that UA may modulate vascular dysfunction, myocardial injury, cardiac fibrosis, and mitochondrial quality control through multiple context‐dependent mechanisms. Importantly, mitochondrial quality control may serve as a central upstream process linking endothelial protection, myocardial metabolic regulation, and cardiac remodeling. Compared with a simple antioxidant interpretation, UA appears to exert broader effects by simultaneously influencing mitochondrial turnover, substrate utilization, and inflammatory signaling. Nevertheless, most current evidence is derived from acute injury or experimental disease models, and whether these mechanisms can modify long‐term cardiovascular outcomes in humans remains unknown.

### 3.3 Neuroprotective Effects

**FIGURE 4:**
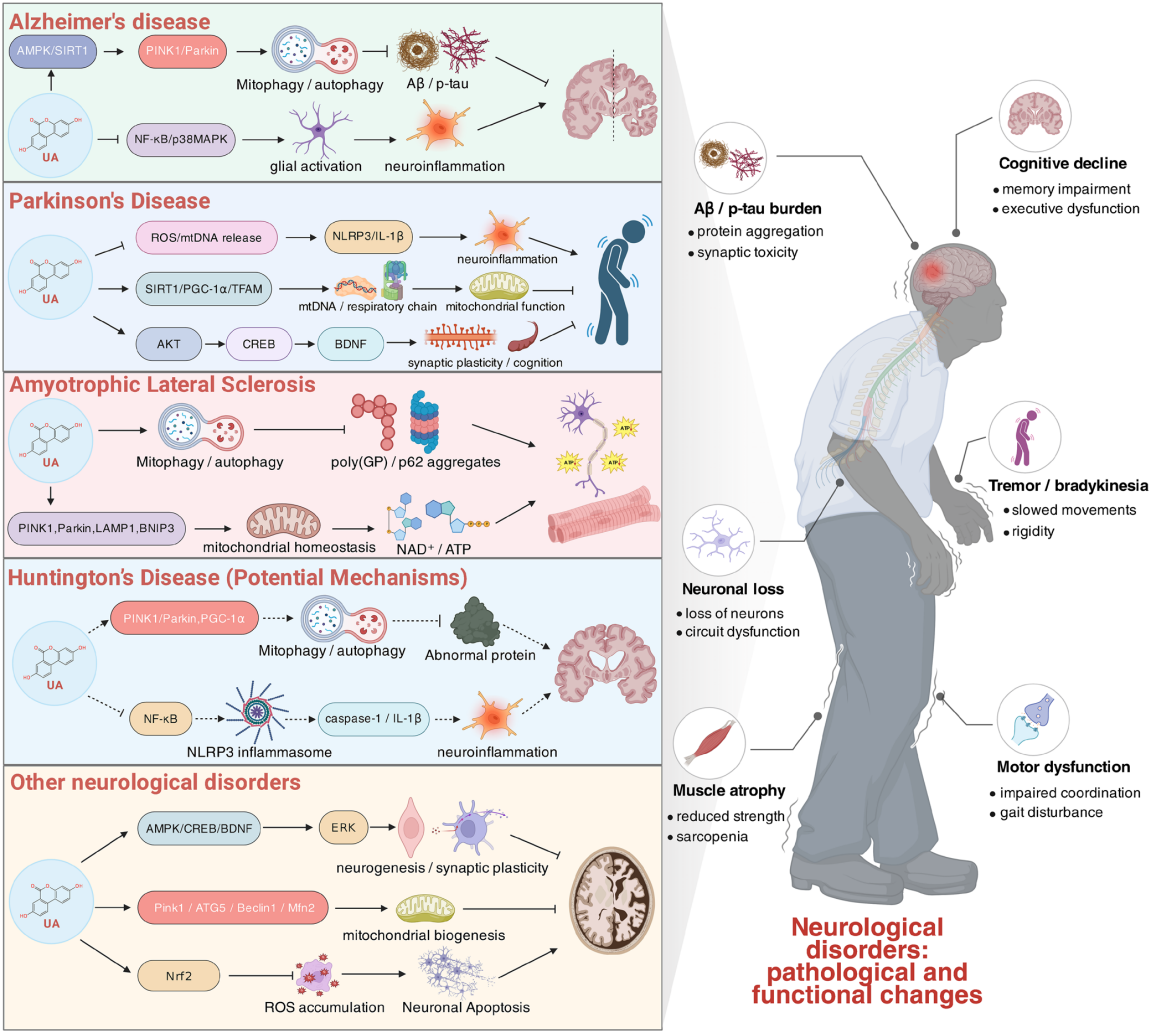
Potential mechanisms of UA in neurodegenerative diseases. This figure summarizes the proposed protective effects of UA in several neurodegenerative diseases, including Alzheimer's disease, Parkinson's disease, amyotrophic lateral sclerosis, Huntington's disease, and other neurological disorders. In Alzheimer's disease, UA may promote AMPK/SIRT1‐ and PINK1/Parkin‐mediated autophagy, thereby reducing Aβ and p‐tau accumulation, and may suppress NF‐κB/p38 MAPK‐related glial activation and neuroinflammation. In Parkinson's disease, UA may reduce ROS/mtDNA release, attenuate NLRP3/IL‐1β‐mediated inflammatory responses, improve mitochondrial function through the SIRT1/PGC‐1α/TFAM pathway, and support synaptic plasticity and cognition via AKT/CREB/BDNF signaling. In amyotrophic lateral sclerosis, UA‐associated autophagy and mitochondrial homeostasis may contribute to reduced poly (GP)/p62 aggregate accumulation and improved NAD^+^/ATP‐related energy metabolism. In Huntington's disease, the illustrated mechanisms remain putative and include regulation of PINK1/Parkin/PGC‐1α‐related autophagy and NF‐κB/NLRP3/caspase‐1/IL‐1β‐mediated neuroinflammation. In other neurological disorders, UA may support neurogenesis and synaptic plasticity through AMPK/CREB/BDNF and ERK‐related signaling, promote mitochondrial biogenesis through pathways involving Pink1, ATG5, Beclin 1, and Mfn2, and reduce ROS accumulation and neuronal apoptosis via Nrf2‐related regulation. The right side of the figure summarizes representative pathological and functional changes associated with neurodegenerative diseases, including Aβ/p‐tau burden, neuronal loss, muscle atrophy, cognitive decline, tremor/bradykinesia, and motor dysfunction. Solid arrows indicate activation or promotion, inhibitory lines indicate suppression, and dashed arrows indicate potential or indirect mechanisms.

Neurological disorders comprise diverse conditions that involve the central and peripheral nervous systems (Dias‐Carvalho et al. 2024; Jiang et al. 2025). As the shared molecular mechanisms of UA have been summarized in the preceding section, this section focuses primarily on disease‐specific neuroprotective evidence. Key findings in neurodegenerative disease models are summarized in Figure 4.

Representative neurodegenerative diseases include Alzheimer's disease (AD), Parkinson's disease (PD), amyotrophic lateral sclerosis (ALS), and Huntington's disease (HD) (Wilson 3rd et al. 2023). Preclinical studies have evaluated UA in several neurodegenerative disease models. In AD models, UA reduced Aβ and p‐tau burdens and improved autophagy–lysosomal function and neuroinflammation, with the involvement of PINK1/Parkin‐mediated mitophagy, the AMPK/SIRT1 axis, Bace1, and NF‐κB/p38MAPK signaling (Gong et al. 2019; Hou et al. 2024). In another AD model, 10 weeks of UA intervention enhanced macroautophagy and Aβ clearance, accompanied by the degradation of LC3 and SQSTM1 (Ballesteros‐Álvarez et al. 2023). In other AD‐related aging models, UA not only reduced oxidative stress and apoptosis in PC12 cells, but also inhibited hippocampal cell apoptosis and GFAP‐positive astrocyte activation in d‐galactose‐induced brain‐aged mice, which was attributed to the regulation of the miR‐34a‐SIRT1/mTOR axis by UA (Chen et al. 2019). In addition, in HT22 cells expressing mutant APP, UA alleviated Aβ‐mediated mitochondrial and synaptic toxicity by enhancing mitochondrial quality control and maintaining synaptic homeostasis (Kshirsagar et al. 2025). Overall, these AD‐related studies suggest that UA may primarily act by restoring mitochondrial quality control and autophagy–lysosomal clearance, thereby indirectly reducing Aβ accumulation, tau pathology, oxidative injury, neuroinflammatory activation, and synaptic dysfunction.

In PD, UA reduced mitochondrial damage and ROS/mtDNA release in microglia, thereby inhibiting NLRP3 inflammasome activation and the production of pro‐inflammatory factors such as IL‐1β, and thus alleviated MPTP‐ and manganese exposure‐related neuroinflammation and dopaminergic neuron loss (Lu et al. 2025; Qiu et al. 2022). In a 6‐OHDA model, UA upregulated the SIRT1/PGC‐1α/TFAM signaling pathway, increased mtDNA copy number, and enhanced the expression of mitochondrial respiratory chain‐related proteins (Liu, Jiang, et al. 2022). Notably, in MPTP‐ and A53T α‐synuclein transgenic mice, UA inhibited the activation of microglia and astrocytes in the hippocampus, reduced inflammation, and enhanced synaptic plasticity by activating the AKT/CREB/BDNF pathway, thereby alleviating PD‐related cognitive impairment (N. Xu et al. 2025). Taken together, these findings indicate that the potential neuroprotective effects of UA in PD models are closely associated with the suppression of mitochondrial damage‐driven innate immune activation, preservation of mitochondrial biogenesis, and maintenance of synaptic plasticity. ALS is a late‐onset disease characterized by the progressive degeneration of upper motor neurons (UMNs) and lower motor neurons (LMNs) (Van Es 2024). In C9orf72‐associated ALS, early autophagy defects are characterized by abnormal aggregation of poly(GP) with SQSTM1/p62. UA promoted the clearance of poly(GP) and damaged mitochondria by activating mitophagy, thereby restoring motor neuron structure and improving motor function (de Calbiac et al. 2024). In copper‐exposed SOD1G93A mice, UA improved ATP/NAD^+^ production by upregulating PINK1, Parkin, LAMP1, and BNIP3, thereby significantly alleviating muscle atrophy, motor dysfunction, and spinal cord neuron loss (Zhang, Gao, Yang, et al. 2025). Direct experimental evidence for UA intervention in HD is currently lacking. Pathways modulated by UA in other disease models, including PINK1/Parkin‐ and PGC‐1α‐related autophagy–lysosomal regulation and NF‐κB/NLRP3‐associated neuroinflammation, overlap with pathological processes implicated in HD. However, the relevance of UA to HD remains hypothetical and requires direct experimental validation (Liu, Jiang, et al. 2022; Qiu et al. 2022; Zhang, Gao, Yang, et al. 2025).

Beyond neurodegenerative diseases, preclinical studies have also examined UA in other neurological disorders. In rat models of cognitive impairment associated with schizophrenia, UA alleviated neuroinflammation by downregulating pro‐inflammatory factors such as TNF‐α, IL‐1β, and IL‐6. At the same time, it upregulated BDNF expression and activated the ERK signaling pathway, thereby promoting hippocampal neurogenesis, reducing dendritic spine loss, and improving memory deficits (Z. Huang et al. 2025). In models of type 2 diabetes and related cognitive impairment, UA alleviated endoplasmic reticulum stress, tau hyperphosphorylation, ROS production, and neuronal apoptosis through the Atp2a3 pathway (Xiao et al. 2023). In addition, UA also alleviated diabetes‐related cognitive impairment by restoring colonic N‐glycan biosynthesis, upregulating tight junction proteins (ZO‐1 and Occludin), and inhibiting TLR4/Myd88 expression (Xiao et al. 2022). In an epilepsy model, UA upregulated ERα and the downstream PGC‐1α/Nrf1/TFAM axis, thereby enhancing mitochondrial biogenesis (Chen et al. 2025). Similarly, in a mouse SD model, UA alleviated neuroinflammation and mitochondrial dysfunction by inhibiting the NF‐κB/NLRP3 inflammasome pathway and restoring the expression of PGC‐1α, PDHA1, ATG5, Beclin1, and Pink1 (Misrani et al. 2024). The UA cyclic ether derivative UAS03, developed to improve stability and bioavailability, suppressed glial activation and activated Nrf2‐related antioxidant signaling in an LPS‐induced mouse model (Maity et al. 2025). In a high‐anxiety model, UA upregulated Mfn2 and mitophagy‐related signaling, improved dendritic–synaptic abnormalities, and restored neuronal circuit activity (Mallet et al. 2025). In cellular and mouse models of depression, the AMPK inhibitor Compound C abolished the antidepressant and neurotrophic effects of UA, supporting a key role for the AMPK/CREB/BDNF signaling axis in its antidepressant‐like effects (Di et al. 2025).

Collectively, the available studies suggest that UA does not target a single disease‐specific pathway but rather modulates several shared hallmarks of neurodegeneration, including mitochondrial dysfunction, impaired autophagy, chronic neuroinflammation, and synaptic deterioration. This broad mechanistic profile may explain why beneficial effects have been reported across multiple neurological disorders. Nevertheless, evidence remains overwhelmingly preclinical, and important translational questions such as blood–brain barrier penetration, optimal dosing, and long‐term neurological safety require further investigation.

### 3.4 Anticancer Properties

Cancer is widely regarded as a complex ecosystem composed of tumor cells and numerous nontumor cells, and it remains a complex and pervasive global health problem (de Visser and Joyce 2023). In recent years, the natural polyphenolic metabolite UA has shown potential anticancer effects in preclinical models of several malignancies, including breast cancer, colorectal cancer, gastric cancer, pancreatic ductal adenocarcinoma (PDAC), and oral squamous cell carcinoma. Current preclinical evidence suggests that the potential anticancer effects of UA may involve inhibition of cancer cell proliferation, induction of apoptosis and autophagy, modulation of the tumor immune microenvironment and gut microbiota composition, and altered responses to selected chemotherapeutic agents.

In preclinical tumor models, UA has been reported to inhibit the proliferation and colony formation of various tumor cells and to induce cell cycle arrest and programmed cell death. In gastric cancer models, UA inhibited cancer cell proliferation and promoted apoptosis by suppressing the PI3K/AKT/mTOR pathway and downregulating the expression of p‐AKT, p‐mTOR, and p‐p70S6K (Zhang, Jiang, et al. 2023). Similar effects were also observed in PDAC cells, in which UA treatment blocked the activation of PI3K/AKT while reducing the phosphorylation levels of its downstream target proteins p70S6K, GSK‐3β, and 4E‐BP1 (Totiger et al. 2019). In addition, UA inhibited cancer cell proliferation by inducing G0/G1‐phase cell cycle arrest and promoted ROS generation in a time‐dependent manner, thereby inducing apoptosis in cancer cells. This effect was associated with Bax upregulation, activation of the caspase cascade, and PARP cleavage, accompanied by increased expression of the autophagy‐related protein LC3‐II and downregulation of p62 (Remadevi et al. 2024). In ER‐positive breast cancer cells, UA was reported to bind to ERα, antagonize 27‐HC, and selectively downregulate proliferation‐related genes such as c‐Myc and PCNA, while showing limited effects on normal cells under the experimental conditions (Vini et al. 2023).

Preclinical studies have also examined the effects of UA on tumor cell migration, invasion, and cytoskeletal remodeling. In gastric cancer models, UA inhibited cancer cell migration and invasion and modulated EMT‐related phenotypes, and this anti‐migratory effect was further enhanced when combined with autophagy inhibitors (Zhang, Jiang, et al. 2023). In a 5‐FU‐resistant colorectal cancer model, UA/UAS03 combined with 5‐FU significantly reduced the migratory capacity of colorectal cancer cells and reversed changes in EMT‐related markers, suggesting that UA may attenuate resistance‐associated migratory phenotypes in this experimental model (Ghosh, Singh, et al. 2022). UA also downregulates the expression of Rac1 and PAK1, induces actin filament disassembly, and triggers cytoskeletal remodeling (Alauddin et al. 2020). In breast cancer models, UA markedly inhibited cancer cell migration and further weakened the invasive capacity of cancer cells by promoting macrophage autophagy, reducing IL‐6 secretion, and maintaining TFEB nuclear translocation (Zheng et al. 2025).

With respect to autophagy and apoptosis, UA has been reported to modulate autophagic and intrinsic apoptotic pathways in different tumor models. In oral squamous cell carcinoma models, UA promoted intracellular ROS accumulation and activated p‐p38 and p‐JNK, thereby inducing Bax‐mediated mitochondrial apoptosis. In addition, ERK inhibitors blocked both apoptosis and autophagy, suggesting that ERK1/2 plays a central role in UA‐induced dual cell death (Remadevi et al. 2024). UA inhibited the Warburg effect in tumor cells by activating Hippo signaling and promoting the phosphorylation‐dependent inactivation of YAP (Qiao et al. 2024).

Preclinical evidence suggests that UA may modulate the tumor immune microenvironment and gut microbiota. In colorectal cancer models, UA induced mitophagy in colonic epithelial cells and CD8^+^ T cells, promoted antigen presentation by tumor epithelial cells, and drove the differentiation of CD8^+^ T cells into memory stem‐like T cells (TSCMs). Through mitochondrial–nuclear signaling pathways involving Pink1, Pgam5, and the Wnt/β‐catenin–PGC‐1α axis, UA maintained T‐cell stemness and was associated with enhanced antitumor T‐cell responses in the experimental models (Denk et al. 2022). Interestingly, UA also induced TSCM‐associated phenotypes in human leukocytes and CAR‐T cells in vitro, supporting further investigation of UA as a potential ex vivo modulatory approach. However, whether this effect can improve the clinical efficacy of CAR‐T‐cell therapy remains to be established (Denk et al. 2022). UA significantly reduced the infiltration of MDSCs, Tregs, and TAMs, suggesting that it can, to some extent, limit immunosuppression in PDAC (Totiger et al. 2019). The Firmicutes/Bacteroidetes (F/B) ratio has been used as a descriptive measure of gut microbial alterations, although its biological interpretation may vary across disease contexts (W. Zhu et al. 2023). In gastric cancer‐bearing mice, UA treatment was associated with changes in gut microbial composition, including reductions in selected potentially cancer‐associated taxa and the F/B ratio (Qiao et al. 2024).

Preclinical studies have explored the potential of UA to modify responses to selected chemotherapy and immunotherapy approaches. UA/UAS03 enhanced sensitivity to 5‐FU by regulating FOXO3‐FOXM1 and downregulating resistance‐associated transporters MRP2 and MRP7 (Ghosh, Singh, et al. 2022). In gastric cancer models, pharmacological inhibition of UA‐associated autophagy with 3‐MA or CQ enhanced its antitumor effect, supporting further preclinical evaluation of this combination. However, the context‐dependent role of autophagy in different tumor microenvironments should be carefully considered before clinical translation (Zhang, Jiang, et al. 2023). In addition, in a pancreatic cancer PKT mouse model, UA monotherapy showed a greater survival benefit than gemcitabine, whereas UA plus gemcitabine did not further improve overall survival in mice. These findings highlight the context‐dependent nature of combination‐based approaches involving UA and indicate that rational treatment combinations should be carefully evaluated before clinical translation (Totiger et al. 2019).

Overall, available preclinical studies suggest that UA may influence several processes involved in tumor development and treatment responses, including cell proliferation, apoptosis, autophagy, metabolic reprogramming, immune regulation, and therapy sensitivity. However, these effects appear to be highly tumor‐ and context‐dependent. Different tumor types and experimental models show distinct responses to UA, indicating that UA is unlikely to function as a universal anticancer agent. More importantly, its anticancer efficacy has not yet been clinically validated, and it remains unclear whether the reported preclinical effects can be achieved at clinically relevant exposures. Future studies should therefore identify tumor settings in which UA‐mediated mechanisms are most likely to be beneficial, clarify optimal dosing and combination strategies, and systematically evaluate safety, potential drug interactions, and therapeutic efficacy in well‐designed clinical studies.

### 3.5 Other Emerging Applications of UA

Beyond the disease contexts discussed above, emerging evidence suggests that UA may also exert protective effects against Clostridioides difficile infection (CDI) and tissue injury associated with environmental toxicants. In a preclinical model of CDI‐induced colitis, UA alleviated colonic inflammation and epithelial injury, restored the expression of tight junction proteins, and reduced *C. difficile* toxin expression without directly inhibiting bacterial growth (S. Ghosh et al. 2024). These findings raise the possibility that UA may serve as an adjunctive approach for limiting toxin‐mediated epithelial damage, although its role alongside standard antimicrobial therapy remains to be established.

Preclinical studies have also explored the protective effects of UA in models of toxicity induced by metals, metalloids, and nanoparticles. In colon epithelial cells and a human intestinal 3D tissue model, UA attenuated trivalent inorganic arsenic‐induced oxidative stress, apoptosis, and barrier dysfunction (S. Ghosh, Banerjee, et al. 2022). In Cr(VI)‐exposed mice, UA mitigated small intestinal injury partly by modulating the PP2A/Hippo/YAP1 pathway (Guo et al. 2024). Taken together, these findings suggest that preservation of intestinal barrier homeostasis may represent a recurring feature of UA‐mediated protection against toxicant‐induced intestinal injury, while the relevant upstream mechanisms may vary across exposure contexts. Beyond the intestinal tract, UA alleviated Cd‐induced hippocampal neuronal and synaptic injury and improved cognitive deficits, partly by suppressing AhR–CYP1A1‐associated mitochondrial reactive oxygen species accumulation and NLRP3‐mediated pyroptosis (Y. Zhu et al. 2026). Similarly, in models of copper oxide nanoparticle exposure, UA promoted the mitophagy‐mediated clearance of damaged mitochondria and mitochondrial reactive oxygen species through a protective pathway involving PINK1 and TAX1BP1, thereby attenuating vascular endothelial injury (Fan et al. 2022). Collectively, these studies point to context‐dependent protective effects involving the maintenance of barrier integrity and the regulation of mitochondrial stress responses, rather than providing evidence that UA acts as a nonspecific antidote to environmental toxicants.

Overall, these emerging studies broaden the potential application spectrum of UA, particularly in toxin‐mediated intestinal injury, neurotoxicity, and vascular endothelial damage. However, the current evidence remains fragmented and is largely limited to cellular, organoid, and animal models. Therefore, these indications should be regarded as hypothesis‐generating research directions rather than near‐term clinical applications. Future studies are needed to determine whether the effective doses used in preclinical models are clinically achievable, and to clarify the long‐term safety, exposure–response relationship, and translational relevance of UA in these emerging contexts.

### 3.6 Potential Effects of UA on Athletic Performance

UA has also been investigated as a potential nutritional strategy to support exercise performance and recovery. As its shared effects on mitochondrial homeostasis, mitophagy, and energy metabolism have been summarized above, this section focuses primarily on exercise‐related outcomes in preclinical models and human studies.

Preclinical studies in nematodes and rodent models suggest that UA may support muscle function and exercise‐related outcomes through multiple mechanisms. Under degenerative stress conditions, UA improved mitophagy, mitochondrial content, muscle strength, and motor performance, with the involvement of PINK1/Parkin‐ and BNIP3‐related pathways (Luan et al. 2021; Ryu et al. 2016; Zhang, Gao, Yang, et al. 2025). In a high‐fat diet‐induced obese mouse model, UA promoted brown adipose tissue thermogenesis and inguinal white adipose tissue browning and increased grip strength, spontaneous activity, and swimming time (B. Xia et al. 2020). UA‐induced skeletal muscle vascularization through SIRT1–PGC‐1α‐related signaling has also been reported in middle‐aged mice (N. Ghosh et al. 2020).

Studies in middle‐aged and older adults provide an initial clinical rationale for athlete‐focused research. UA supplementation was associated with reduced plasma acylcarnitine levels and favorable changes in selected measures of muscle function and physical performance, including lower‐limb strength, walking endurance, peak oxygen uptake, and fatigue resistance (Andreux et al. 2019; Liu, D'Amico, et al. 2022; Singh et al. 2022b). However, these studies were conducted in nonathlete populations and should not be interpreted as direct evidence of ergogenic effects.

Evidence regarding UA supplementation in trained populations remains limited, although several recent studies have begun to evaluate its effects on exercise performance and recovery. In resistance‐trained participants with more than 3 years of training experience, UA supplementation increased maximal isometric contraction strength and repetitions to failure and was accompanied by changes in biomarkers related to protein turnover, inflammation, oxidative stress, and AMPK‐ and PPARγ‐related signaling (Zhao et al. 2024). In elite endurance runners, UA supplementation appeared to be more closely related to training adaptation and recovery than to short‐term performance enhancement. UA upregulated pathways related to mitochondrial protein complexes and reduced creatine kinase levels within 24 h after exercise, without altering maximal mitochondrial respiratory function. Under short‐term, high‐intensity training camp conditions at high altitude, UA significantly upregulated molecular pathways related to mitochondrial protein complexes in skeletal muscle without altering maximal mitochondrial respiratory function, and significantly reduced creatine kinase levels within 24 h after exercise (Whitfield et al. 2025). In youth soccer players, UA supplementation alongside conventional training was associated with improvements in Yo‐Yo Intermittent Recovery Test performance and countermovement jump height (Monsalve Acevedo et al. 2025).

Collectively, current evidence supports further investigation of UA as a potential sports nutrition strategy, but firm conclusions regarding its ergogenic effects remain premature. Existing studies are limited in number and vary substantially in participant characteristics, exercise modalities, intervention durations, and outcome measures. Notably, improvements in metabolic and mitochondrial biomarkers appear to be more consistent than changes in conventional performance outcomes. This pattern suggests that UA may act primarily as a metabolic resilience‐ or recovery‐supporting supplement rather than as a direct ergogenic aid. Future larger randomized controlled trials should clarify whether specific populations, such as older adults, athletes undergoing intensive training, or individuals with impaired mitochondrial function, are more likely to benefit from UA supplementation.

## 4 Bioavailability, Safety, and Human Clinical Evidence of UA

### 4.1 Gut Microbiota Variability and Urolithin Metabotypes

The endogenous production of UA depends on the capacity of the gut microbiota to convert dietary ellagitannins (ETs) and ellagic acid (EA) into urolithins. Human intervention studies have consistently identified three urolithin metabotypes (UMs), which differ not only in the amount of UA produced but also in their terminal urolithin profiles. Individuals with metabotype A (UM‐A) produce UA as the main terminal urolithin, whereas those with metabotype B (UM‐B) generate UA together with isourolithin A and/or urolithin B. By contrast, metabotype 0 (UM‐0) individuals do not produce detectable levels of intermediate or terminal urolithins following the intake of ET‐ or EA‐rich foods (Cortés‐Martín et al. 2024; Tomás‐Barberán et al. 2014). These metabotypes have been observed across different dietary sources, including pomegranate, walnuts, and berries (Tomás‐Barberán et al. 2014). However, their relative distribution is not uniform across populations. A large cohort study involving individuals aged 5–90 years suggested that aging is an important factor associated with the distribution of UMs, while studies comparing normoweight and overweight‐obese individuals have also linked metabotype patterns to host metabolic status and gut microbial ecology (Cortés‐Martín et al. 2018; Selma et al. 2016). Thus, the intake of an equivalent amount of ETs or EA does not necessarily result in comparable UA exposure across individuals.

Importantly, UMs may also help explain the heterogeneous responses observed in dietary intervention studies. In overweight‐obese individuals, pomegranate extract supplementation did not significantly improve cardiovascular risk biomarkers when participants were analyzed as a single group. However, stratification according to UM revealed a dose‐dependent improvement in several blood lipid parameters specifically in UM‐B individuals (González‐Sarrías et al. 2017). More recently, UA production was associated with the effects of pomegranate extract on the gut microbial metabolism of bile acids and cholesterol in individuals with mild dyslipidemia. Reductions in fecal bile acids and coprostanol were correlated with urolithin concentrations, particularly fecal UA, whereas comparable changes were not observed in UM‐0 individuals (Cortés‐Martín et al. 2024). Taken together, these findings suggest that UMs should be considered functional gut‐microbial signatures rather than merely descriptive classifications. Incorporating metabotype stratification into future studies may help distinguish variability arising from precursor conversion from variability in the biological response to UA itself, thereby supporting more precise nutritional strategies. Whether UMs can also predict individual responses to direct UA supplementation remains unclear and warrants prospective investigation.

Direct UA supplementation may provide a practical strategy to reduce the variability associated with microbiota‐dependent precursor conversion. However, it may not completely eliminate interindividual differences, because subsequent absorption, metabolism, tissue distribution, and clearance may still influence systemic exposure and biological responses.

### 4.2 Pharmacokinetics and Clinical Achievability of Experimental Doses

In a randomized crossover study of healthy adults, only approximately 40% of participants substantially converted pomegranate‐derived precursors into UA, whereas supplementation with 500 mg UA resulted in more consistent circulating levels and over sixfold greater exposure to UA and its conjugates than pomegranate juice (A. Singh et al. 2022a). Following absorption, UA rapidly undergoes extensive phase II metabolism and therefore circulates predominantly in conjugated forms. The most common UA metabolites in plasma are UA‐glucuronide and UA‐sulfate, whereas the level of free parent UA is low and is usually difficult to detect (García‐Villalba et al. 2022; Singh et al. 2022a). These metabolites generally reach peak plasma concentrations 4–8 h after administration, with a Tmax of approximately 6 h, and exhibit relatively long half‐lives of approximately 17–22 h. In some studies, the half‐lives of certain sulfated metabolites have exceeded 25–50 h (Kuerec et al. 2024). In addition, human skeletal muscle analyses, experimental studies, and physiologically based pharmacokinetic modeling suggest that the effects of UA are not restricted to circulating biomarkers and may extend to peripheral tissues (Aichinger et al. 2023; Andreux et al. 2019; Ryu et al. 2016). However, the strength of evidence differs across tissues, ranging from direct measurements in experimental models and human skeletal muscle to model‐based predictions. These findings provide a pharmacological basis for the potential tissue‐level actions of UA.

The translational relevance of preclinical findings depends on whether the experimental doses and concentrations are realistically achievable in humans. In the first‐in‐human phase 1 study, single ascending doses of 250, 500, 1000, and 2000 mg UA were evaluated, while repeated daily doses of 250, 500, and 1000 mg were administered for 28 days (Andreux et al. 2019). Notably, the lowest clinical dose of 250 mg/day was selected on the basis of preclinical efficacy observed with oral administration of 50 mg/kg/day UA in mice (Andreux et al. 2019; Ryu et al. 2016). Subsequent randomized clinical trials further showed that oral supplementation with 500–1000 mg/day UA for up to 4 months is feasible in middle‐aged adults, while 1000 mg/day has also been administered for 4 months in older adults (Liu, D'Amico, et al. 2022; Singh et al. 2022b). Thus, 500–1000 mg/day represents a supplementation range that has already been evaluated in human intervention studies. However, this does not imply that all doses used in experimental models can be directly extrapolated to humans. A systematic review of in vivo studies identified substantial heterogeneity in the doses, routes of administration, and intervention periods used across animal models (Tow et al. 2022). Differences in species‐specific metabolism, dosing schedules, administration routes, and tissue exposure limit direct comparisons based solely on nominal doses.

The interpretation of in vitro studies requires even greater caution. Human intervention studies indicate that free parent UA is present at relatively low circulating concentrations, whereas conjugated metabolites, particularly UA‐glucuronide, predominate in plasma (Andreux et al. 2019; García‐Villalba et al. 2022; Singh et al. 2022a). Consistent with this pattern, physiologically based pharmacokinetic modeling predicted low‐nanomolar peak concentrations of free UA in most tissues following postbiotic supplementation (Aichinger et al. 2023). Under a repeated dose scenario of 1000 mg/day, the predicted peak concentrations of free UA were approximately 5.0 nM in muscle, 9.9 nM in brain tissue, and 7.6 nM in liver, while the predicted concentration in gut tissue was higher but remained in the nanomolar range (Aichinger et al. 2023). The same modeling study suggested that concentrations at which several beneficial or toxic effects have previously been observed in vitro are unlikely to be reached in vivo (Aichinger et al. 2023). Therefore, experiments using micromolar concentrations of free UA should primarily be viewed as mechanistic evidence rather than as direct representations of clinically achievable exposure.

Importantly, this exposure gap does not exclude biological activity in humans, because clinical trials have reported changes in mitochondrial biomarkers and selected functional outcomes at orally achievable doses (Andreux et al. 2019; Liu, D'Amico, et al. 2022; Singh et al. 2022b). Instead, it raises the possibility that sustained low‐level exposure, conjugated metabolites, or tissue‐specific processes such as local deglucuronidation may contribute to the observed effects (Aichinger et al. 2023). Future studies should integrate pharmacokinetic and pharmacodynamic analyses, direct tissue‐exposure measurements, and dose–response comparisons to define the clinically relevant exposure window of UA.

### 4.3 Safety and Tolerability of UA

Available preclinical and clinical evidence suggests that UA has a generally favorable short‐ to medium‐term safety and tolerability profile. In rats, a systematic safety assessment of orally administered synthetic UA included genotoxicity assays, toxicokinetic analyses, and repeated‐dose studies. UA was not genotoxic in the overall test battery. Moreover, dietary administration for 28 and 90 days did not produce alterations in clinical parameters, blood chemistry, or hematology, and no target‐organ toxicity or specific toxic mechanisms were identified. In the 90‐day study, the no‐observed‐adverse‐effect level (NOAEL) was the highest dose tested, corresponding to 5% UA by weight in the diet, or 3451 mg/kg body weight/day in males and 3826 mg/kg body weight/day in females (Heilman et al. 2017).

Human studies have provided additional evidence of tolerability at orally achievable doses. In a first‐in‐human phase 1 study involving healthy sedentary older adults, single ascending doses of 250–2000 mg and repeated daily doses of 250–1000 mg for 28 days were evaluated. Serious adverse events were not observed, and no nonserious adverse events were considered product‐related, and no clinically relevant abnormalities were observed in liver or kidney function, hematology, or urinalysis (Andreux et al. 2019). Longer randomized trials subsequently reported favorable tolerability with 1000 mg/day for 4 months in older adults and with 500–1000 mg/day for 4 months in middle‐aged adults (Liu, D'Amico, et al. 2022; Singh et al. 2022b). More recently, a randomized trial in 50 healthy middle‐aged adults receiving 1000 mg/day for 28 days recorded four adverse events in the UA group and five in the placebo group, with no significant changes in kidney function parameters or liver enzymes (Denk et al. 2025).

Nevertheless, the available evidence remains preliminary. A systematic review identified only five human studies involving 250 healthy participants, with intervention periods ranging from 28 days to 4 months and no postintervention follow‐up (Kuerec et al. 2024). In addition, although physiologically based pharmacokinetic modeling suggests that potentially genotoxic concentrations observed in vitro are unlikely to be reached systemically, local intestinal exposure and possible endocrine‐related effects warrant further investigation (Aichinger et al. 2023). Therefore, current evidence supports favorable short‐ to medium‐term tolerability rather than definitive long‐term safety. Larger and longer trials are required, particularly in patients with chronic diseases, individuals with impaired organ function, and populations receiving concomitant medications.

### 4.4 Human Clinical Evidence of UA

At present, clinical evidence for UA in humans is gradually accumulating, but it remains concentrated in specific fields. Available clinical studies have mainly focused on healthy aging and physical fitness, particularly muscle function, exercise performance, fatigue recovery, and molecular mechanisms related to mitochondrial function (Table 1). Studies showed that long‐term oral administration of UA improved indices of muscle function, such as lower limb muscle strength, muscle endurance, walking distance, and peak VO_2_, in the absence of additional exercise intervention, and these improvements were accompanied by favorable changes in inflammatory markers and biomarkers related to mitochondrial metabolic function.

**TABLE 1:** Clinical research findings of UA.

Author	Year	Disease/Population	Intervention/Dosage	Outcomes	Type	References
Andreux	2019	Sedentary older adults	500–1000 mg/day for 4 weeks	This study first demonstrated that oral UA was safe and well tolerated, and induced improvements in mitochondrial and cellular health.	UA supplementation RCT	Andreux et al. (2019)
Singh	2022	Middle‐aged overweight adults (muscle aging)	500–1000 mg/day for 4 months	Improved muscle strength and walking performance, enhanced mitochondrial metabolic function, and reduced CRP levels.	UA supplementation RCT	Singh et al. (2022b)
Liu	2022	Older adults (reduced muscle endurance)	1000 mg/day for 4 months	Improved muscle endurance and reduced acylcarnitine and inflammatory marker levels.	UA supplementation RCT	Liu, D'Amico, et al. (2022)
Zhao	2024	Male resistance‐trained athletes	1 g/day for 8 weeks	Increased maximal voluntary isometric contraction strength and repetitions to failure, and reduced oxidative stress and inflammatory responses.	UA supplementation RCT	Zhao et al. (2024)
Denk	2025	Middle‐aged adults with immunosenescence (45–70 years)	1000 mg/day for 4 weeks	Improved T‐cell metabolism and immunosenescence‐related phenotypes, and promoted immune remodeling.	UA supplementation RCT	Denk et al. (2025)
Whitfield	2025	Highly trained endurance runners	1000 mg/day for 4 weeks	Increased VO2max and reduced CK‐related damage markers, modulated complex‐related pathways, and promoted recovery.	UA supplementation RCT	Whitfield et al. (2025)
Jarrard	2021	Localized prostate cancer (active surveillance)	Pomegranate fruit extract (PFE) for 1 year	Showed good safety and tolerability, increased urolithin metabolites, and reduced oxidative stress levels.	Translational evidence (extract)	Jarrard et al. (2021)
Istas	2018	Healthy adults (vascular endothelial function)	Red raspberry intake (a source of ellagitannins)	UA metabolites were significantly associated with improved flow‐mediated dilation (FMD).	Translational evidence (metabolite)	Istas et al. (2018)
Kaplan	2022	Individuals with abdominal obesity (at risk of brain atrophy)	Green‐MED dietary intervention for 18 months (with increased UA levels)	Increased UA levels were strongly associated with attenuated hippocampal atrophy.	Translational evidence (metabolite)	Kaplan et al. (2022)
Napier	2025	Healthy adults (UA‐producing capacity)	Synbiotic intervention for 91 days	Reduced systemic inflammation, as reflected by decreased CRP levels.	Translational evidence (microecology)	Napier et al. (2025)

In older adults, although no significant differences were observed in systemic exercise capacity indices, such as the six‐minute walk test, due to factors such as placebo effects or individual variability, UA still showed advantages in more sensitive indices of local muscle fatigue. Clinical trials in the field of immunosenescence further showed that UA may promote the remodeling of immune phenotypes and metabolic capacity, as reflected by the enhancement of CD8+ T cell‐related features. It is also noteworthy that, in short‐term clinical studies involving elite athletes, the effects of UA on competition outcomes or athletic performance were not consistent; however, its beneficial effects on postexercise muscle damage and recovery‐related indices were generally more consistent, suggesting that UA may serve as a potential nutritional strategy to support exercise adaptation and recovery in the future. It should be emphasized that the current research landscape of UA extends far beyond human clinical trials. A large number of animal and cell studies demonstrated that UA has potential benefits in cancer, degenerative musculoskeletal diseases, neurological disorders, and cardiovascular diseases. Its mechanisms of action mainly involve mitochondrial quality control, regulation of inflammation and oxidative stress, remodeling of immune responses, and maintenance of apoptosis/autophagy network homeostasis. In summary, the current clinical translation of UA in humans represents only part of its multisystem potential, and its clinical research value in other disease areas remains substantial. Therefore, high‐quality clinical trials targeting specific patient populations are still needed in the future to clarify the optimal dose, population stratification, long‐term safety, and the durability of benefits of UA.

## 5 Current Limitations and Future Perspectives

With UA research shifting from mechanistic studies to translation, critically defining current evidence gaps and future directions is paramount for this gut microbiota‐derived metabolite. First, most mechanistic and disease‐related findings remain derived from in vitro studies and animal models. These studies have provided important insights into mitophagy, oxidative stress, inflammatory signaling, autophagy, apoptosis, and immune regulation; however, the doses, exposure patterns, disease models, and intervention windows used in experimental systems may not fully reflect human physiological conditions. Therefore, the molecular mechanisms identified in preclinical studies should be interpreted as putative and context‐dependent rather than as established therapeutic mechanisms in humans.

Human clinical evidence for UA also remains limited in scale, duration, and clinical scope. Trials to date have mainly focused on healthy aging, skeletal muscle function, exercise‐related outcomes, mitochondrial biomarkers, inflammatory markers, and short‐ to medium‐term tolerability. Although these studies suggest potential benefits in selected metabolic and functional outcomes, most have involved relatively small sample sizes, selected populations, and intervention periods of only several weeks to a few months. Differences in participant characteristics, dosage regimens, outcome measures, and intervention contexts further limit cross‐study comparability and make it difficult to determine whether UA produces consistent effects across broader populations. In particular, evidence supporting disease‐modifying effects in cardiovascular, neurological, musculoskeletal, or cancer‐related conditions remains largely preclinical, and disease‐oriented randomized controlled trials with clinically meaningful endpoints are still lacking.

Interindividual variability represents another major challenge for UA translation. Endogenous UA production depends on the gut microbiota‐mediated conversion of ellagitannins and ellagic acid, and individuals differ in their urolithin metabotypes, including UM‐A, UM‐B, and UM‐0. Moreover, circulating UA is mainly present as phase II conjugated metabolites, whereas free UA remains at relatively low levels. These factors raise important questions regarding bioavailability, active molecular forms, tissue exposure, and responder identification. Future studies should therefore integrate microbiota profiling, metabotype classification, pharmacokinetic analysis, and biomarker‐based evaluation to clarify the relationship between UA exposure and biological response.

Long‐term safety also remains insufficiently defined. Although available studies suggest that oral UA is generally well tolerated over short‐ to medium‐term interventions, evidence regarding chronic supplementation, long‐term efficacy, and safety in older adults, patients with chronic diseases, and individuals receiving multiple medications remains limited. Future trials should include larger sample sizes, longer follow‐up, standardized safety monitoring, and assessment of potential drug–nutrient interactions.

Nevertheless, these limitations also highlight important future opportunities. UA may be particularly valuable in precision nutrition, healthy aging, muscle function maintenance, exercise recovery, and metabolic resilience, where mitochondrial quality control and metabolic adaptation are central biological processes. Future studies could explore personalized supplementation strategies based on urolithin metabotypes, combine UA with exercise training or dietary interventions, and develop formulations with improved bioavailability and tissue exposure. In addition, multi‐omics approaches, including gut microbiome analysis, metabolomics, proteomics, and mitochondrial function profiling, may help identify responders and clarify clinically relevant mechanisms. Well‐designed multicenter randomized controlled trials with standardized doses, population stratification, clinically meaningful endpoints, and extended follow‐up will be essential to determine whether the promising mechanistic and biomarker‐level effects of UA can be translated into durable, safe, and clinically meaningful benefits in humans.

## 6 Conclusion

UA is a key gut microbiota‐derived metabolite generated from ellagic acid and ellagitannins, and has attracted increasing attention because of its potential relevance to aging‐related functional decline and multisystem health. Evidence summarized in this review suggests that UA may influence pathological processes involved in degenerative musculoskeletal diseases, cardiovascular diseases, neurological disorders, and cancer, mainly through mechanisms related to mitochondrial quality control, inflammatory regulation, oxidative stress, autophagy, and cell survival. Among these mechanisms, mitophagy and mitochondrial homeostasis appear to represent central regulatory nodes that connect several signaling pathways, including Nrf2, AMPK, SIRT1, PI3K/Akt, and NF‐κB.

Beyond disease‐related contexts, UA also shows potential as a nutritional strategy for supporting skeletal muscle function, exercise adaptation, mitochondrial metabolic efficiency, and postexercise recovery. However, available evidence suggests that its benefits may be more closely related to metabolic resilience and recovery support than to direct short‐term enhancement of athletic performance. Although preliminary clinical trials indicate that oral UA is generally well tolerated over short‐ to medium‐term interventions and may improve selected health‐related outcomes, current human evidence remains limited in scale, duration, population diversity, and disease‐specific endpoints. Therefore, UA should be regarded as a promising but still clinically unconfirmed candidate for nutritional and translational applications. Further large‐scale, long‐term, disease‐oriented randomized controlled trials incorporating population stratification, clinically meaningful endpoints, and biomarker‐based evaluation are needed to determine its efficacy, optimal dose, target populations, long‐term safety, and translational value.

## Author Contributions

**Zijiang Yang:** data curation, formal analysis, visualization, writing – original draft. **Chenggen Guo:** writing – review and editing. **Ziao Deng:** writing – review and editing. **Xinxuan Xue:** supervision, writing – review and editing.

## Funding

This work was supported by Youth Fund for Humanities and Social Sciences Research of the Ministry of Education, 24YJC890016. Key Project of Philosophy and Social Sciences Research of the Hubei Provincial Department of Education, 24D100. Youth Project of Science and Technology Research of the Hubei Provincial Department of Education, Q20234103.

## Ethics Statement

Ethical approval was not required because this review was based solely on previously published literature and did not involve human participants, animals, identifiable personal data, or clinical samples.

## Conflicts of Interest

The authors declare no conflicts of interest.

## Data Availability

No new datasets were generated in this review. The data that support the findings of this study are available from the corresponding author upon reasonable request.
